# A Virtual Screening Approach to Evaluate the Multitarget Potential of a Chalcone Library with Binding Properties to Oligopeptidase B and Cysteine Proteinase B from *Leishmania (Viannia) braziliensis*

**DOI:** 10.3390/ijms26052025

**Published:** 2025-02-26

**Authors:** Patrícia Queiroz Monteiro, Edgar Schaeffer, Alcides José Monteiro da Silva, Carlos Roberto Alves, Franklin Souza-Silva

**Affiliations:** 1 Laboratório de Biologia Molecular e Doenças Endêmicas, Instituto Oswaldo Cruz, Fundação Oswaldo Cruz, Avenida Brasil 4365, Manguinhos, Rio de Janeiro 21040-900, RJ, Brazil; queiroz.biotec@gmail.com; 2 Laboratório de Catálise Orgânica, Instituto de Pesquisas de Produtos Naturais Walter Mors, Universidade Federal do Rio de Janeiro, Avenida Carlos Chagas Filho 373—Bloco H, Cidade Universitária, Rio de Janeiro 21941-599, RJ, Brazil; edgar.ippn@gmail.com (E.S.); alcides@ippn.ufrj.br (A.J.M.d.S.); 3 Laboratório de Modelagem de Sistemas Biológicos, Centro de Desenvolvimento Tecnológico em Saúde, Instituto Oswaldo Cruz, Fundação Oswaldo Cruz, Avenida Brasil 4036, Manguinhos, Rio de Janeiro 21040-361, RJ, Brazil; franklin.frankss@gmail.com; 4 Laboratório de Pesquisa Pré-Clínica, Universidade Iguaçu, Avenida Abílio Augusto Távora 2134, Dom Rodrigo, Nova Iguaçu 26260-045, RJ, Brazil

**Keywords:** *Leishmania (Viannia) braziliensis*, proteinases, chalcones, multitarget inhibitors, molecular docking

## Abstract

Leishmaniasis remains a significant public health problem in Brazil, particularly due to *Leishmania (Viannia) braziliensis*, which is associated with severe dermatological syndromes. The current treatments are limited by toxicity and uncertain efficacy, highlighting the need for new compounds with pharmacological potential. This study investigates chalcones as multitarget binding agents for oligopeptidase B (OPB) and cysteine proteinase B (CPB), which are critical pathogenic determinants of *L. (V.) braziliensis*. The methodology involved replacing methoxy groups with aryl motifs at various positions within the chalcone structures and introducing specific functional groups at the C-4 position. This was followed by a virtual screening approach using molecular docking to assess interactions with the target proteinases. Several chalcones from the virtual library (*n* = 178) exhibited high binding affinities for OPB and CPB, outperforming control ligands. A total of 30 chalcones with multitarget potential were identified, with fluorinated compounds C-191 and C-135 emerging as promising inhibitors, distinguished by the best energy rankings for both enzymes. ADMET analyses confirmed the viability of these chalcones as drug candidates, with most adhering to Lipinski’s rules. These data suggest that chalcones may provide new multitarget treatment options for leishmaniasis.

## 1. Introduction

*Leishmania* spp. are parasites widely distributed across the Americas, Africa, and Asia. Sand flies act as vectors for these parasites, which can infect mammalian hosts, including humans. Within these hosts, the protozoa can cause clinical manifestations characterized by cutaneous leishmaniasis (CL) and visceral leishmaniasis (VL). Approximately 700,000 to 1 million new cases of leishmaniasis are reported worldwide annually [1]. In Brazil, both clinical forms have been documented, with several *Leishmania* spp. identified, contributing to an endemic scenario marked by unique features relevant to public health, particularly in rural areas and increasingly in urban centers [2]. Notably, among the species associated with CL in Brazil, *Leishmania (V.) braziliensis* is the prominent causative agent of American cutaneous leishmaniasis (ACL), a dermatozoonosis with high endemicity that poses significant treatment challenges and represents a major public health concern [3,4].

Several treatment options are available for VL and CL, which are selected based on the characteristics of the infections. The current treatment approaches rely on two main therapeutic options: (i) first-line drugs, such as sodium stibogluconate (Pentostan™) and n-methylglucamine antimoniate (Glucantime™), which are associated with considerable cardiotoxicity during the treatment and uncertainties regarding their efficacy; and (ii) second-line drugs, including pentamidine and amphotericin B, which are challenging to administer and carry toxicity risks [5]. Treatment strategies vary depending on the form of the disease. For CL cases resistant to standard treatments, amphotericin B may be considered. First-line treatments for VL typically include liposomal amphotericin B and miltefosine. Mucocutaneous leishmaniasis (ML) shares treatment options similar to those for CL, such as systemic antimonials, amphotericin B, and miltefosine, although therapy may need to be more aggressive due to the complexity of the disease [1,2,3,4,5].

Pharmacologically innovative initiatives for leishmaniasis treatment remain limited. However, additional strategies are being explored, including drug combinations aimed at mitigating side effects and improving treatment efficacy. For example, the combination of miltefosine and paromomycin, both administered orally, has shown effectiveness in treating VL [6,7]. Another promising combination therapy involves amphotericin B and liposomal paromomycin, which has proven effective for treating CL [8]. Furthermore, new compounds are being investigated for their leishmanicidal activity against both VL and CL.

Despite these efforts, no new drugs with a satisfactory therapeutic index and low toxicity have been developed [9]. Among the numerous challenges in treatment through conventional pharmacology, it is essential to highlight the multifactorial nature of the pathology, the uncertainty surrounding parasite elimination—which may lead to infection relapse—and the adaptive capabilities of the pathogen [10,11].

Considering the complex modulation network between *Leishmania* spp. and the diseases they cause in mammalian hosts, proteinases are recognized as important enzymes and emerge as promising candidates for a multitarget treatment approach due to their involvement in various biochemical pathways [12]. In *Leishmania* spp., these enzymes play a crucial role in pathogenicity by facilitating the invasion and survival of the parasite within the vertebrate host. They degrade components of the extracellular matrix and immune system proteins, aiding in the evasion of the immune response [12,13,14]. Additionally, enzymes such as oligopeptidase B (OPB) and cysteine proteinase B (CPB), which can alter cell signaling, promoting the spread of infection, are part of the organization of protease genes in the genome of *Leishmania* spp. [12,15]. A growing debate has arisen regarding the potential role of *Leishmania* spp. proteinases in modulating Th1/Th2 immune responses through degrons in immune system proteins [16]. Therefore, proteinases are essential to the life cycle of *Leishmania* spp. and the pathogenesis of the diseases they cause.

Given the critical roles of OPB and CPB in parasite virulence and survival, this study aims to provide new insights into their potential as therapeutic targets, particularly in *Viannia* subgenus parasites, such as *L. (V.) braziliensis*, where they remain underexplored. By focusing on these less-studied targets, this study seeks to contribute valuable data to the development of novel treatment strategies for leishmaniasis.

The multitarget approach focusing on proteinases is particularly relevant for addressing leishmaniasis, which is a complex disease with multiple factors contributing to its pathogenesis. Targeting various physiological pathways of the parasite is a viable strategy to enhance treatment effectiveness by impeding parasite proliferation with greater specificity and efficiency. In drug development, there has been a shift from a single-target approach to a multitarget paradigm in designing new compounds. This trend is underscored by an analysis of drugs approved by the Food and Drug Administration (FDA), which identifies small molecules as multitarget drugs for therapies, including combination therapies [17,18]. Over time, the field of polypharmacology, which encompasses both multitarget and combined chemotherapies, has been growing [19,20]. These attributes, characterized by metabolic directionality, present increasingly promising and safer avenues as chemotherapeutic approaches for multifactorial diseases [21], such as leishmaniasis.

In this context, understanding the binding characteristics of proteinases—particularly focusing on natural compounds such as chalcones—can enhance the range of compounds available for drug development. Chalcones are natural organic compounds extracted from various plant species, such as *Piper methysticum* and *Handroanthus* spp. This group of compounds has garnered attention in drug discovery and development as potential therapeutic agents for neurodegenerative, cardiovascular, metabolic, inflammatory diseases, diabetes, cancer, fungal infections, bacterial infections, viral infections, and protozoan diseases [22,23,24]. The diverse spectral properties of these compounds significantly contribute to the potential expansion of the chalcone library, based on novel chemical structures. Additionally, several studies suggest that chalcones could be employed in a multitarget strategy for treating multifactorial diseases due to their specificity for different classes of enzymes. The chemotherapeutic potential of natural and synthetic chalcones against leishmaniasis has been proposed [25,26,27]. This includes flavokawain B (FKB), a trans-chalcone with a hydroxy group at position 2′ and methoxy groups at positions 4′ and 6′ [28], as well as C020, a 2′-methoxy-4′-fluoro-chalcone bearing an aryl group [29].

Other chalcones have demonstrated selective activity against *Leishmania (L.) amazonensis* and *Leishmania (L.) infantum* through in vitro and in vivo approaches [30,31,32,33,34,35]. The potential of a synthetic chalcone has also been successfully assessed against *Leishmania* spp., suggesting its action on cytosolic tryparedoxin peroxidase activity [36]. Evidence of leishmanicidal activity was reported for pinostrobin chalcone, isolated and identified as effective in a hamster-*L. (V.) braziliensis* model via topical application of an ointment containing 6% of this compound on the animal’s dorsum [27].

The present study was inspired by the therapeutic potential of FKB [27] and the aryl-chalcone C020 [28], which exhibit antileishmanial activity for *L. (L.) amazonensis* and *L. (L.) major*. A potential target for aryl-chalcones is proteinases, as their activity has already been evaluated against the cysteine proteinase of *Plasmodium falciparum* [37].

The binding profile of chalcones to serine and cysteine proteinases of *L. (V.) braziliensis* is explored in this study, proposing a multitarget approach to simultaneous binding to the catalytic site of OPB (amino acid residues: serine, aspartic acid, and histidine) and CPB (amino acid residues: glutamine, cysteine, histidine, and asparagine) of this parasite. The objective is to evaluate the ability of chalcones to interact with targets such as CPB and OPB, based on molecular docking rankings that apply a consensus approach for both enzymes. Additionally, favorable absorption, distribution, metabolism, excretion, and toxicity (ADMET) properties were assessed by using a comprehensive, free online prediction platform, suggesting their potential as prototype drug candidates for prototype drugs. Notably, evidence indicates that arylated chalcone derivatives may serve as effective inhibitors of CPB and OPB in *L. (V.) braziliensis*, highlighting their multitarget potential.

## 2. Results and Discussion

In drug development, the design of new compounds is shifting from a single-target model to a multitarget approach. This strategy is particularly relevant for leishmaniasis, as these are multifactorial diseases, as targeting multiple biochemical pathways within the parasite effectively enhances treatment efficacy. This group of compounds has garnered attention in drug discovery and development as potential therapeutic agents for neurodegenerative, cardiovascular, metabolic, and inflammatory diseases, as well as diabetes, cancer, fungal, bacterial, and viral infections, and diseases caused by protozoa, enhancing treatment efficacy. Thus, the search for novel compounds with multitarget profiles, capable of interfering with the parasite’s multiplication with greater specificity and efficiency is ongoing, with strong support for the therapeutic potential of chalcones. In this context, our study explores structural modifications in chalcones to enhance their multitarget potential.

**Figure 1:**
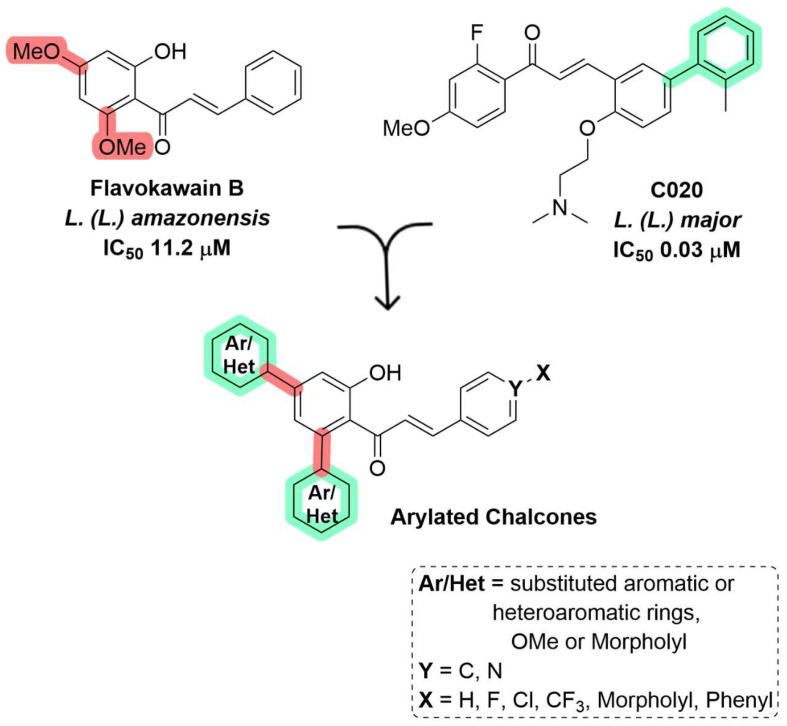
Rational design of arylated chalcones. The methoxy moiety (red) in flavokawain B was substituted by aryl groups (green), creating a new pool of arylated chalcones.

Therefore, among a set of 178 distinct arylated chalcones (Supplementary Figure S1 and Table S1), the focus was on replacing methoxy groups with aryl groups at various positions within the structure of FKB and C020. Certain functional groups were introduced at the chalcone C-4 center (Figure 1).

By incorporating various aryl and heteroaryl rings with electron-donating or electron-withdrawing properties, as well as lipophilic and basic groups, the aim was to enhance the interactions of these chalcones with CPB and OPB proteinases and to create a new virtual library based on these compounds. The substitution patterns were rationalized to investigate their potential effects on enzymatic activity, serving as a strategy for designing proteinase inhibitors for *Leishmania* spp. A similar approach has been proposed to facilitate the development of novel lead candidates for antimalarial treatments [37].

Afterward, the potential of these new chalcone library compounds as lead candidates for inhibitors of *L. (V.) braziliensis* proteinase was assessed. Given that both proteinases constitute the predominant classes of proteinases encoded in the genome of *Leishmania* spp. and are described as essential contributors to virulence [14,16], this study proceeded with evaluations of the ligand affinities for these enzymes through molecular docking. The structural model of each target was generated using the SwissModel server. The sequences of the parasite proteinases were first compared with their respective templates, 2XE4 for OPB and 6P4E for CPB, showing identities of 85.5% for the first and 76.5% for the latter, indicating a reliable template to follow in the modeling assays (Supplementary Figure S2). The predicted models were validated by the SAVES web server, with Ramachandran plots indicating the residues within the most favored regions for OPB and CPB (Supplementary Figure S3). The Root Mean Square Deviation (RMSD) values between the template and the homology model structures were 0.763 Å and 0.251 Å, respectively. These data show highly successful homology models, as they have RMSD values ≤ 2 Å. Docking validation was performed by measuring the RMSD of redocked native crystal ligands for both enzymes. Furthermore, CPB was complexed with the control inhibitor aza-nitrile inhibitor (named GES) showing satisfactory values (RMSD: 1.2 Å and −8.107 kcal/mol), and OPB was complexed with antipain (RMSD: 1.6 Å and −7.427 kcal/mol).

Furthermore, the interactions of commercial inhibitors (trans-epoxysuccinyl-L-leucylamido(4-guanidino)butane: E-64; phenylmethylsulfonyl fluoride: PMSF) with their respective proteinases were evaluated: E-64 (RMSD: 1.6 Å and −8.0 kcal/mol, for CPB) and PMSF (RMSD: 2.0 Å and −6.59 kcal/mol, for OPB). To assess the potential of chalcone derivatives on the proteinases of *L. (V.) braziliensis*, two comparison parameters were utilized. A set of compounds with better affinities was identified, ranging from −9.29 to −7.38 kcal/mol for OPB and −9.09 to −7.13 kcal/mol for CPB (Supplementary Table S2). The compounds’ occupancies of the enzyme catalytic sites mirrored those of crystallized structures from the Protein Data Bank (PDB). Notably, the chalcones displayed more effective and specific binding, as evidenced by their higher docking scores (Supplementary Table S2).

This approach evaluated the potential of chalcones using the results of virtual screening, and their respective controls are presented, detailing the energy score values for the 178 compounds, along with their respective highest and lowest affinities. The best-ranked energy score values were associated with OPB for chalcone 132 (−9.29 kcal/mol) and with CPB for C-128 (−9.02 kcal/mol) (Supplementary Table S2).

**Figure 2:**
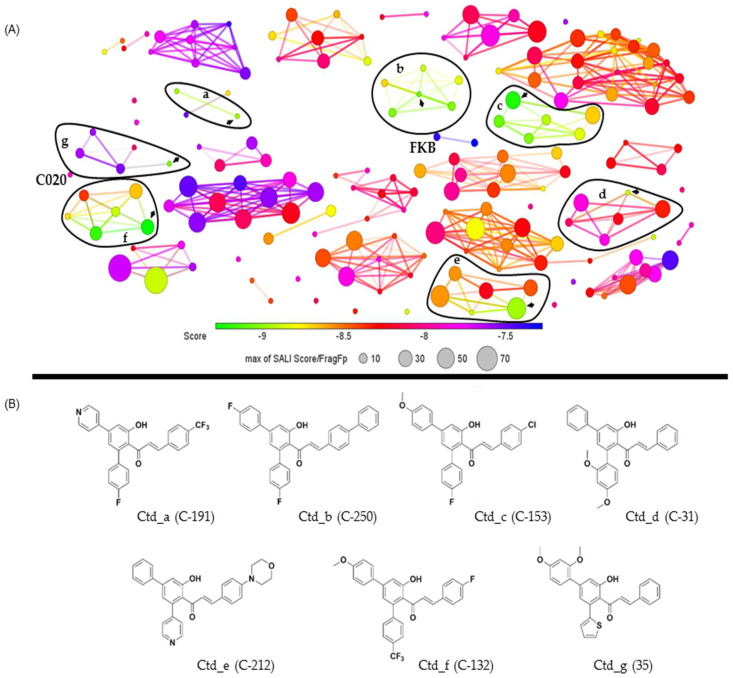
The clustering analysis of *Leishmania (Viannia) braziliensis* OPB affinities and chalcone structural similarities is presented. The graph illustrates the clustering of chalcones (**A**), emphasizing selected subclusters, with their respective centroids indicated by arrows (a, b, c, d, e, f, and g). The chemical structures of the chalcones are depicted, representing the centroids of the clusters along with their respective binding energy values (−8.97 _ a, −9.07 _ b, −9.22 _ c, −8.90 _ d, −8.97 _ e, −9.29 _ f, −9.04 _ g, and −8.99 _ h) (**B**). The circle sizes indicate structural similarity (ranging from 10% to 70%), determined using the fragment dictionary-based binary fingerprint (FragFp) ratio as the structure–activity landscape index (SALI), with values exceeding 70%. The circle colors represent the molecular docking score range (−9.29 kcal/mol to −7.5 kcal/mol). Additionally, the rationally designed precursors from the chalcone library, FkB and C020, were incorporated into the clustering analysis, as illustrated in the figure.

**Figure 3:**
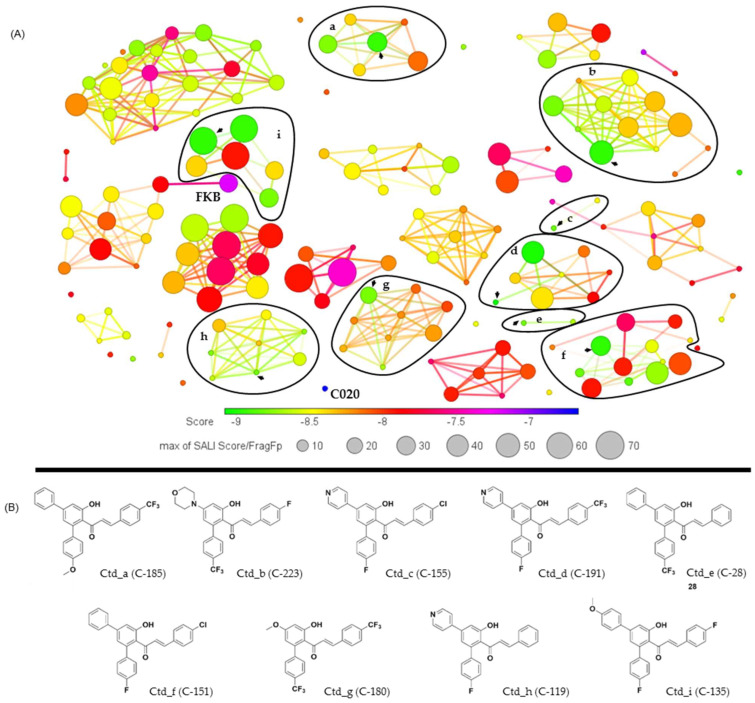
The clustering analysis of *Leishmania (Viannia) braziliensis* OPB affinities and chalcone structural similarities is presented. The graph illustrates the clustering of chalcones (**A**), emphasizing selected subclusters, with their respective centroids indicated by arrows (a, b, c, d, e, f, and g). The chemical structures of the chalcones are depicted, representing the centroids of the clusters along with their respective binding energy values (−8.95 _ a, −8.97 _ b, −8.83 _ c, −9.09 _ d, −8.81 _ e, −8.97 _ f, −8.80 _ g, −8.81_h e −8.97 _ i) (**B**). The circle sizes indicate structural similarity (ranging from 10% to 70%), determined using the fragment dictionary-based binary fingerprint (FragFp) ratio as the structure–activity landscape index (SALI), with values exceeding 70%. The circle colors represent the molecular docking score range (−9.29 kcal/mol to −7.5 kcal/mol). Additionally, the rationally designed precursors from the chalcone library, FkB and C020, were incorporated into the clustering analysis, as illustrated in the figure.

The clustering analysis of chalcones was related to the proteinase affinities of *L. (V.) braziliensis* in this phase of this study. Correlations between the structures and energy values were performed using the DataWarrior software. Clusters were formed based on the virtual screening scores and the similarities of the aromatic ring core systems in these compounds (Supplementary Table S3). Subsequently, the clusters were presented according to the highest affinities between chalcones and the analyzed proteinases, with selection based on the best-ranked energy value of each group (Figure 2 and Figure 3).

The components of the chalcone/OPB clusters were selected (*n* = 7) based solely on the energy score values (−9.29 to −8.97 kcal/mol), defining the chalcone with the highest docking energy score as the centroid (Ctd) for each cluster with specific modifications (Figure 2). Cluster ‘a’–Ctd_191 featured modifications in ring 1 [R1 (phenyl or pyridinyl) and R2 (4-F/4-CF3-phenyl)], while ring 2 had modifications (CF3 or H) at position X. Cluster ‘b’–Ctd_250 exhibited modifications in ring 1, with R1 (4-H/4-F-phenyl) and R2 (4-H/4-F/2,4-Me-phenyl), while ring 2 featured F, Cl, phenyl, or H substituents at position X. Cluster ‘c’–Ctd_153, with modifications in ring 1 R^1^ (4-H/4-OMe-phenyl) and R^2^, presented aromatic groups with H, F, and methoxy at the 4 position, while ring 2 featured F, Cl, or H substituents at position X. Cluster ‘d’ –Ctd_31 presented modifications in ring 1 R^1^ (MeO- or phenyl) and R^2^ (2-OMe/2,4-OMe/3,4-OMe-phenyl) except for chalcone 10 and 128, which showed a 2-OMe-naphthyl group, while ring 2 showed the substituents (F or H) at position X. Cluster ‘e’–Ctd_212, in general, the chalcones of this group presented modifications in ring 1 R^1^ (4-H/4-F/4-OMe-phenyl or pyridinyl) and R^2^ (4-OMe/4-F-phenyl or pyridinyl), while ring 2 featured morpholyl at position X. Cluster ‘f’—Ctd_132, the chalcones of this group presented ring 1 in R^1^ (4-H/4-OMe-phenyl) and R^2^ (4-F/4-OMe/4-CF_3_-phenyl), while the ring 2 modification showed substituents (F, CF_3_, H, or Cl) at position X. Cluster ‘g’–Ctd_35, the components of the group presented substituent ring 1 with R^1^ (MeO- or 2,4-OMe-phenyl), and R^2^ (thiophenyl), while ring 2 had (F, Cl, or H) at position X or N at the Y position.

The structure and energy variations between the clusters require additional comments. Clusters ‘b’ and ‘c’ showed more homogeneous inter-cluster score values (−9.07 kcal/mol to −8.70 kcal/mol and −9.22 kcal/mol to −8.69 kcal/mol, respectively). Clusters ‘a’ and ‘b’ exhibited lower structural similarity between groups (≤10%), while cluster ‘e’ contained chalcones with higher similarity (≥30%). Lastly, clusters ‘d’ and ‘g’ displayed the greatest variation in inter-cluster scores (−8.90 kcal/mol to −7.83 kcal/mol and −9.04 kcal/mol to −7.53 kcal/mol, respectively) (Supplementary Table S1).

The chalcone/CPB clusters (*n* = 9) were defined by the docking energy score range (−9.02 to −8.80 kcal/mol), with the chalcone featuring the best energy centroid (Ctd) for each cluster (Figure 3). Cluster ‘a’—Ctd_185 featured modifications in ring 1 R1 (4-H/4-OMe-phenyl) and R^2^ (4-F/4-OMe/4-CF_3_-phenyl), while ring 2 had modifications at the X position (H/F/CF_3_/Cl). Cluster ‘b’—Ctd_223 featured modifications in ring 1 R^1^ (4-OMe-phenyl, MeO-, or morpholyl) and R^2^ (4-H/4-F/4-CF_3_/4-OMe-phenyl, or pyridinyl), while ring 2 showed modifications at the Y position (N) or at the X position (F/CF_3_/Cl/H/morpholyl). Cluster ‘c’—Ctd_155 had modifications in ring 1 R^1^ (4-F-phenyl, pyridinyl, MeO-, or morpholyl) and R^2^ (4-F-phenyl, pyridinyl, furanyl, or thiophenyl), while ring 2 showed modifications at the X position (H/Cl/F/morpholyl) and Y position (N). Cluster ‘d’—Ctd_191 had modifications in ring 1 R1 (4-H/4-F-phenyl, MeO-, or pyridinyl) and R2 (4-F/2-OMe/2,4-OMe/3,4-OMe-phenyl, 2-OMe-naphthyl, or pyridinyl), while ring 2 showed modifications at the X position (H/F/CF_3_). Cluster ‘e’—Ctd_28 featured modifications in ring 1 R^1^ (phenyl) and R^2^ (4-F/4-CF_3_-phenyl), while ring 2 showed modifications at the X position (H/CF3). Cluster ‘f’—Ctd_151 had modifications in ring 1 R1 (4-H/4-F/4-OMe/2,4-OMe-phenyl, or MeO-) and R^2^ (4-H/4-F/4-OMe/2,4-Me-phenyl, MeO-, or thiophenyl), while ring 2 showed modifications at the X position (phenyl/F/H/Cl) and Y position (N). Cluster ‘g’–Ctd_180 featured modifications in ring 1 R^1^ (MeO-) and R2 (4-H/4-F/4-CF_3_/4-Me/4-OMe-phenyl, or 2-OMe-naphthyl), while ring 2 showed modifications at the X position (H/F/Cl/CF3). Cluster ‘h’– Ctd_119 had modifications in ring 1 R^1^ (4-H/4-F-phenyl, or pyridinyl) and R2 (4-F-phenyl, or pyridinyl), while ring 2 showed modifications at the X position (H/F) and Y position (N). Cluster ‘i ’—Ctd_135 featured modifications in ring 1 R^1^ (4-H/4-OMe-phenyl, or MeO-) and R^2^ (4-H/4-F/4-OMe-phenyl, or MeO-), while ring 2 showed modifications at the X position (H/F/Cl).

Furthermore, among this set of chalcone/OPB clusters, Ctd_168 needs additional consideration. Despite showing one of the better docking energy scores (−8.94 kcal/mol), it was not included as part of a cluster with other chalcones. This chalcone featured modifications in ring 1, with R^1^ (4-OMe-phenyl) and R^2^ (4-CF_3_-phenyl), while ring 2 had modifications at the Y (N) position.

Additionally, /mol. Finally, clusters **‘c’** and **‘e’** showed the lowest similarity among the evaluated clusters ‘a’ and ‘b’ showed more heterogeneous inter-cluster variations in similarity, while cluster ‘i’ displayed more homogeneous inter-cluster characteristics. However, all these clusters exhibited energy values ranging from −8.97 kcal/mol to −7.92 kcal/mol. The chalcones in clusters ‘g’ and ‘h’ show a similarity of ≤20% among themselves, and both exhibited minimal energy variation (−8.81 kcal/mol to −8.04 kcal/mol). Similarly, the compounds in clusters ‘d’ and ‘f’ presented a similarity of ≤40% and exhibited an energy variation of ≥ −1.00 kcalgroups, with slight energy variations (−8.83 kcal/mol to −8.45 kcal/mol) (Supplementary Table S1).

**Figure 4:**
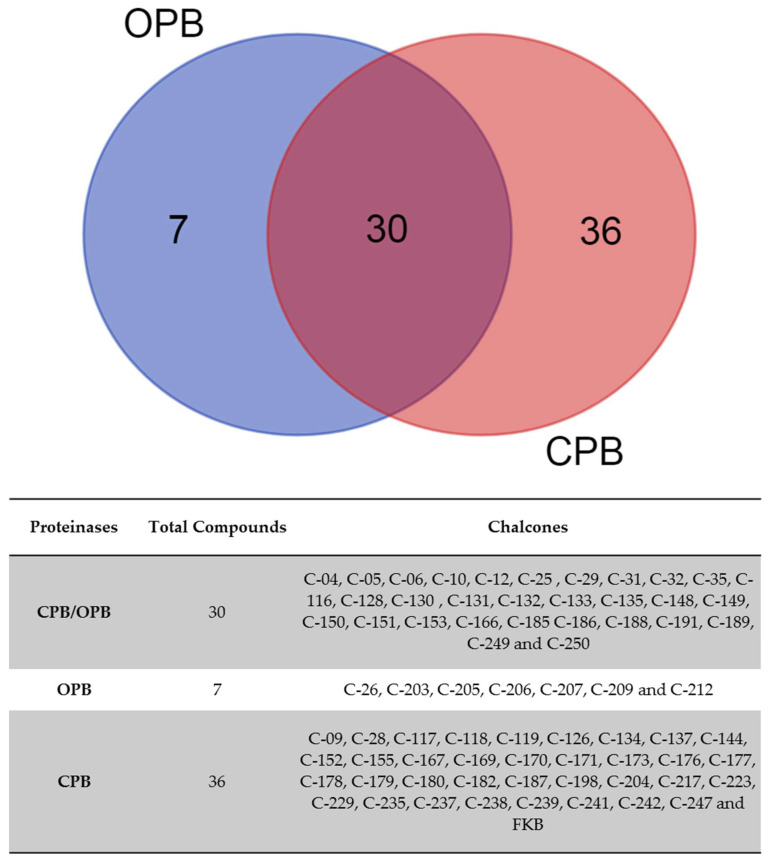
Identification of potential multitarget chalcones through overlap analysis. Chalcone clusters were subjected to Venn analysis, a classic data visualization tool that illustrates the logical relationships between chalcone sets based on the respective energies. The diagram and table depict the specificity of each protease (OPB: *n* = 7 chalcones; CPB: *n* = 36 chalcones) and the overlap between OPB and CPB (*n* = 30 chalcones).

After these analyses, the chalcones from the clusters for OPB and CPB were submitted to a Venn diagram approach to provide a diagrammatic representation based on clusterization. Considering the Venn diagram (Figure 4), the majority of chalcones specifically interacted with either CPB (*n* = 36) or OPB (*n* = 7) (Figure 4). The intersection showed chalcones with multitarget activity (*n* = 30), indicating their binding potential to both enzyme classes and, consequently, their involvement in distinct virulence pathways of the parasite. Furthermore, the docking energy score ranking (Supplementary Table S2) also highlights the distinctive affinity characteristics of these compounds for OPB and CPB proteinases.

A comparative analysis of the binding energy of the chalcones present in the intersections of the Venn analyses highlights a group of clustered molecules for both enzymes. Based on the energy criterion, two chalcones stood out with the best energy rankings for both enzymes: C-191 (OPB: −8.97 kcal/mol; CPB: −9.09 kcal/mol) and C-135 (CPB: −8.97 kcal/mol; OPB: −9.09 kcal/mol). Both chalcones exhibited high binding affinities, outperforming the control ligands, although C-132 was not a centroid in the cluster for CPB. Thus, based on these analyses, it is pertinent to propose this multitarget approach, as demonstrated by the commercial inhibitors E-64 and PMSF [38,39].

The selection of chalcone compounds with multitarget potential, based on the docking energy score cutoff of the control inhibitor was the right decision since it enhanced the possibility of the chalcones forming complexes with both proteinases due to their affinity for each inhibitor enzyme. This relevance is underscored by the fact that the catalytic mechanism of both proteinases involves one or more tetrahedral transition states. A critical aspect of their catalytic function is the oxyanion hole, which contains hydrogen bond donors that stabilize the transition state through interactions with the tetrahedral oxyanion. The hydrogen bonds formed in the transition state are likely stronger than those in the Michaelis complex, thus favoring the stabilization of the transition state over the ground state and enhancing the overall catalytic efficiency [40].

**Figure 5:**
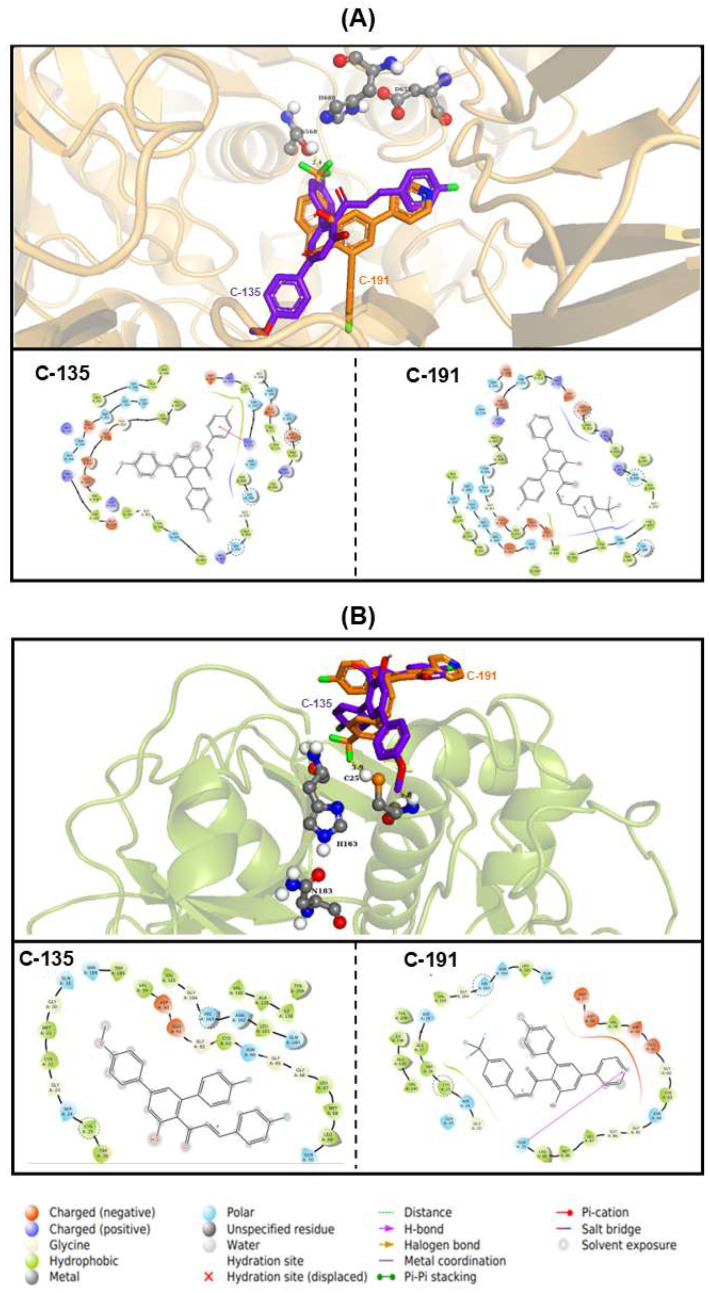
Molecular interactions of chalcone derivatives with *Leishmania (V.) braziliensis* proteinases. Docking assay for (**A**) oligopeptidase B (OPB) and (**B**) cysteine proteinase B (CPB) complexed with chalcones C-135 and C-191. The interactions between chalcone derivatives and proteases are represented in both three-dimensional (3D) and two-dimensional (2D) structures. In the 3D representations, chalcones and catalytic site amino acids are shown as stick models, while proteins are represented as cartoons. In the 2D representations, the chemical structures of the chalcones are displayed, with catalytic site amino acids highlighted by dotted circles for both enzymes.

Based on the docking results, this study proceeded with an analysis of the molecular interactions of the multitarget chalcones that exhibited the best binding energy, evaluating the details of these interactions at the catalytic site of both proteinases (Figure 5) compared with their crystallographic references 2XE4 and 6P4E, respectively (Supplementary Figure S4). The studied proteinases revealed interaction profiles with the crystallized inhibitors (GES and antipain) and were compared with the interactions of the chalcones. Chalcones C-135 and C-191 remained close to the catalytic residues of OPB and CPB, with distances ranging from 2.4 to 4.8 Å (Figure 5A,B).

The crystallographic structure 6p4e (CPB) showed a predominance of hydrophobic residues (64%), polar residues (12%), and negatively charged residues (4%). In contrast, the structure 2xe4 (OPB) exhibited a predominance of hydrophobic residues (51%), polar residues (18%), negatively charged residues (12%), and positively charged residues (10%) (Supplementary Figure S4). Both chalcones, C-191 and C-135, revealed similar interaction profiles for serine and cysteine proteinase; however, specific differences in the interaction modes were noted.

The interaction results for the best affinity score, C-135 for OPB, indicated a pre-dominance of hydrophobic residues (35%) at the catalytic site, followed by polar residues (27%), negatively charged residues (16%), and positively charged residues (14%). Additionally, π–cation interactions between lysine 211 (LYS211) and the fluorinated groups in R^2^ were detected. The interactions of C-191 with the catalytic site of this enzyme also showed a predominance of hydrophobic residues (39.1%), followed by polar residues (30.4%), negatively charged residues (17.8%), and positively charged residues (8.7%) (Figure 5A).

Regarding CPB, the best affinity score in the molecular docking assay was observed with C-191, whose interaction results with the catalytic site showed a predominance of hydrophobic residues (45%), followed by polar residues (26%), negatively charged residues (13%), and a hydrogen bond with glycine 70 (GLN70). Additionally, hydrogen bonds were detected between GLN70 and the nitrogenated groups in R^1^. For this enzyme, the analysis also showed that C-135 interacted with a predominance of hydrophobic residues (50%), followed by polar residues (25%) and negatively charged residues (6%) (Figure 5B).

For drug development, it is crucial to evaluate specific predictive physicochemical properties that are aligned with ADMET analyses. This enables the filtering of candidate compounds according to Lipinski’s rules, a widely used strategy in computer-aided drug discovery and design [41]. Data for the analysis of absorption, distribution, metabolism, excretion, and toxicity predictions for the selected molecules were obtained using the Swiss-ADME and pkCSM web servers (http://www.swissadme.ch/, accessed on 29 July 2024). This approach involved 178 aryl chalcones, which were categorized based on the number of Lipinski’s rules violations: no violations, one violation, and two violations. Thus, 70% of chalcones did not violate any rules, 28% violated just one, and 4% violated three parameters (Supplementary Table S4).

Moreover, chalcones characterized by multitarget potential were identified as having no violations (C-10, C-35, C-04, C-166, C-05, C-130, C-148, C-06, and C-12), one violation (C-191, C-128, C-151, C-135, C-185, C-131, C-116, C-133, C-28, C-149, C-189, C-187, C-250, C-249, C-153, C-132, C-32, C-31, C-25, and C-29), and two violations (C-186 and C-150) of Lipinski’s rules, (Supplementary Table S4). In this context, all the potential multitarget compounds described here complied with at least three of the parameters outlined in Lipinski’s rules. It is important to highlight that several candidates that have become commercial drugs also exhibit exceptions to Lipinski’s rules. Nonetheless, their application remains essential in the selection and strategic planning of biologically active molecules [42].

Over the years, discussions have emphasized the concepts and applications of drugs initially considered single-target agents that were later discovered to have multiple-target actions in treating various diseases [42,43]. This evolution is particularly relevant in the field of polypharmacology, which encompasses multitarget and combination therapies, offering increasingly promising and safer chemotherapeutic approaches for multifactorial diseases such as leishmaniasis [17]. In this context, the advantages of in silico methods were explored here to identify polypharmacological inhibitors based on arylated chalcones.

Although this study provides evidence of the in silico drug design strategy as a powerful tool for reducing the number of ligands, it is recommended that this novel panel of aryl chalcones be validated in experimental assays with CPB and OPB proteinases. The findings presented here introduce the multitarget potential of 30 novel arylated chalcone derivatives, highlighting C-191 and C-135 as potential inhibitors for OPB and CPB based on their binding properties. Both novel chalcones, characterized by fluorinated substituents, are predicted to exhibit enhanced biological effects through metabolic pathways involving the CPB and OPB proteinases of *L. (V.) braziliensis*, positioning them as valuable candidates for the treatment of ACL.

Furthermore, the findings for both C-191 and C-135 presented here align with the trend of FDA-approved fluorinated drugs. The number of these compounds has steadily increased over the past two decades, driven by the success of fluorinated corticosteroids and fluoroquinolones [44]. Fluorine’s high electronegativity and small atomic size enhance drug potency, selectivity, metabolic stability, and pharmacokinetics. Its substitution has been widely studied to improve biological activity and stability in drug development [45].

## 3. Materials and Methods

### 3.1. Chalcone Library

The chemical design of the chalcone structures was based on their synthetic convenience and on a set of reactions that were described previously [46]. The chalcone molecules were designed using the software PerkinElmer ChemDraw^®^ Professional, version 22.2.0.3300.

### 3.2. Molecular Modeling and Validation

The sequences of oligopeptidase B (OPB–LbrM_09.0850) and cysteine protease B (CPB–LbrM_08.0830) from *Leishmania* (*V*.) *braziliensis* were retrieved from TrypTripDB (https://tritrypdb.org/, accessed on 12 July 2024) and subsequently modeled using Swiss-Model (https://swissmodel.expasy.org/). The homology models were generated using the crystallographic structures 6P4E (Leishmania mexicana CPB in complex with an aza-nitrile inhibitor) and 2XE4 (oligopeptidase B from *Leishmania major* in complex with an antipain inhibitor), obtained from the Protein Data Bank (https://www.rcsb.org/, accessed on 15 July 2024). The validation process was performed using SAVES (https://saves.mbi.ucla.edu/, accessed on 29 July 2024) [47].

The ligands were designed based on FKB and C020 modified to incorporate to aromatic rings to increase the binding area with the enzyme’s pocket. These modifications were applied to every derivative of these scaffolds by adding halogen or nitrogen groups. Subsequently, energy minimization was conducted using the MMF94s force field in Avogadro 3.0. The molecular docking studies were performed using the Dockthor server (https://www.dockthor.lncc.br/v2/, accessed on 5 August 2024). Docking was performed under rigid-body conditions for the ligand, with the addition of hydrogen atoms. The following parameters were validated and applied for both serine and cysteine classes: for OPB, the central atom coordinates (*x* = −6 Å, *y* = 25 Å, and *z* = 73 Å) and the box dimensions (*x* = 12 Å, *y* = and 15 Å, *z* = 16Å); for CPB, the central coordinates (*x* = 2.28 Å, *y* = 13.83 Å, and *z* = 47.07 Å) and the box dimensions (*x* = 15 Å, *y* = 15 Å, and *z* = 15 Å). Finally, the binding mode analysis was conducted using the interaction mode diagrams generated in Schrödinger Maestro version 13.4.134. A radius of 7 Å was defined to analyze the receptor/ligand interactions.

### 3.3. Chalcone Clustering

The chalcone molecules were clustered using the software DataWarrior V6.1.0 (https://openmolecules.org/help/chemistry.html). Chalcone clustering was performed based on docking score affinities and ring system similarity. The variation between chalcones was determined using the scaffold structure method, and clusters were selected based only on the energy score values, defining the chalcone centroid (Ctd). This clustering approach was applied to each enzyme studied. To define the clusters, a cutoff point of −0.32 kcal/mol was established based on the largest difference between the energy score values of the leading compounds in the ranking and the energy of the chalcones defined as multitarget. The structural similarity data of the chalcones were represented as a fragment dictionary-based binary fingerprint ratio (FragFp), demonstrating the structure–activity landscape index (SALI). These analyses were based on the original publication that combines dynamic graphical views and interactive filtering with chemical intelligence [48].

### 3.4. Comparative Analysis of Chalcone Overlap

The chalcones in the clusters, based on the centroids, were analyzed using the Bioinformatics and Evolutionary Genomics service (https://bioinformatics.psb.ugent.be/webtools/Venn/, accessed on 9 September 2024) to calculate the intersections of the chalcone lists. This service produces graphical outputs in Scalable Vector Graphics (SVG) and Portable Network Graphics (PNG) formats. The textual output indicates which chalcones are part of the intersection (OPB/CPB) or are unique to OPB or CPB.

### 3.5. Administration, Distribution, Metabolism, Excretion, and Toxicity

To assess the administration, distribution, metabolism, excretion, and toxicity (ADMET) profile according to Lipinski’s rule of five, their pharmacokinetics and physicochemical parameters were evaluated using the Swiss-ADME (http://www.swissadme.ch/, accessed on 23 September 2024) and the pkCSM (https://biosig.lab.uq.edu.au/pkcsm/, accessed on 24 September 2024) web servers.

## 4. Conclusions

The evidence presented here demonstrates that chalcones exhibit potential multitarget activity against *L. (V.) braziliensis* proteinases. This finding is particularly relevant for compounds C-191 and C-135, which showed better affinities for the OPB and CPB proteinases, respectively, as observed through our in silico analysis. While there is evidence of multitarget binding potential for OPB and CPB, it is essential to advance to the next stages of chemical synthesis, efficacy testing, and safety evaluations, as recommended for preclinical studies, to establish these compounds as multitarget drugs. Furthermore, adherence to good synthesis practices is necessary to ensure the high quality of these compounds. Based on the results obtained, we propose that these candidate compounds are in the early stages of the drug development process for the treatment of ACL.

## Data Availability

The original contributions presented in this study are included in the article/Supplementary Material. Further inquiries can be directed to the corresponding author(s).

## Supplementary Materials

The following supporting information can be downloaded at https://www.mdpi.com/article/10.3390/ijms26052025/s1.
