# Supplementary material for: A Virtual Screening Approach to Evaluate the Multitarget Potential of a Chalcone Library with Binding Properties to Oligopeptidase B and Cysteine Proteinase B from Leishmania (Viannia) braziliensis
Source: Int J Mol Sci. 2025 Feb 26;26(5):2025. doi: 10.3390/ijms26052025 (PMC11900450; doi:10.3390/ijms26052025)
Supplement: Supplementary file 1 [file ijms-26-02025-s001.zip › ijms-3446655-supplementary.pdf]

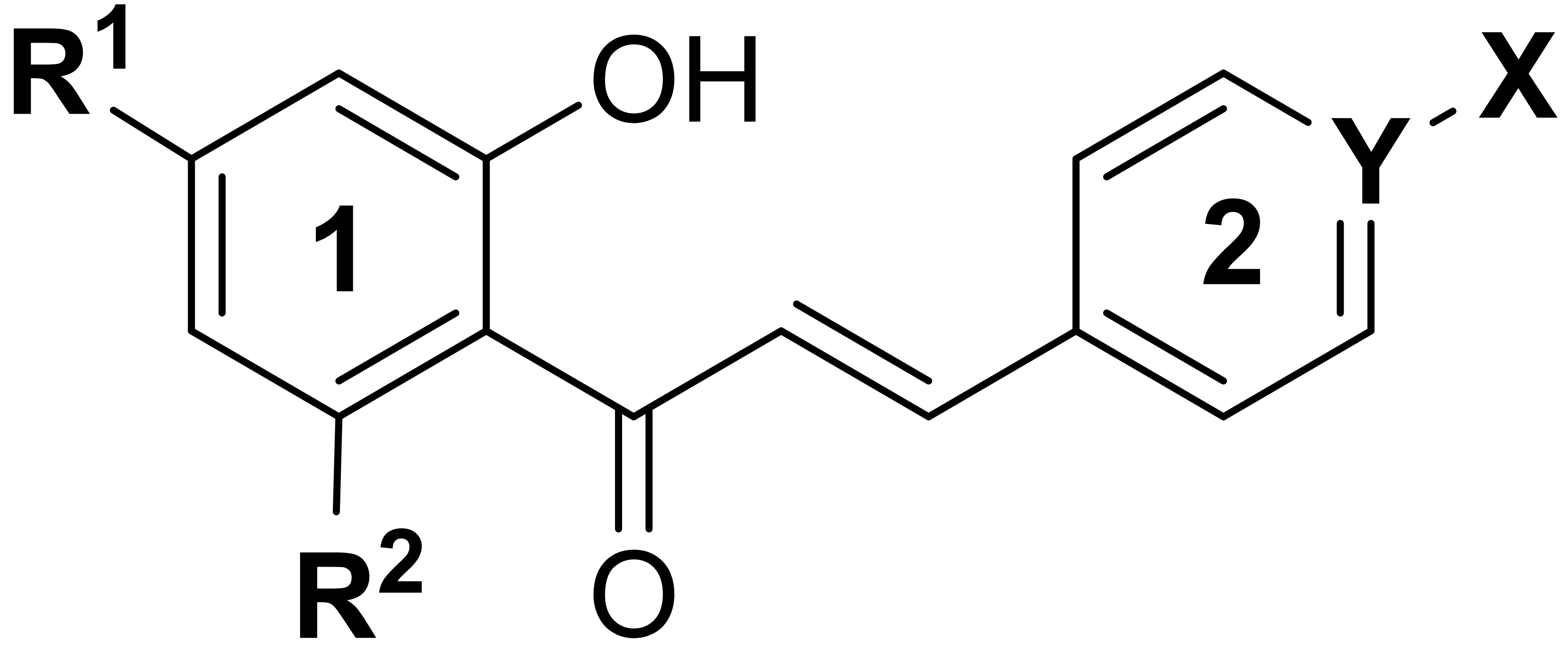

Figure S1. Two-dimensional structure of the chalcone core. The rings (aromatic or heteroaromatic rings) and moiety (MeO or Morpholyl) were placed in R^1 and R^2 positions of chalcone structures.

Table S1. Combinatorial analysis of mono- and diarylated chalcones bearing different substituents and/or heteroaromatic rings: Incorporating various aryl and heteroaryl rings with electron-donating or withdrawing, lipophilic, and basic groups has been proposed to enhance enzymatic interactions and create a new virtual library of chalcones. The substitution patterns are being rationalized to study their effects on enzymatic activity. This table shows the incorporation of MeO and morpholyl moieties (✓).

Compound (ID)*	Rings									
	**1**								**2**	
	**R**[1]				**R**[2]				**Y**	**X**
	Aromatic	Heteroaromatic	OMe	Morpholyl	Aromatic	Heteroaromatic	OMe	Morpholyl		
1			✓		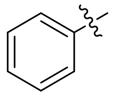				C	H
2			✓		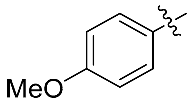				C	H
3			✓		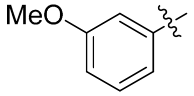				C	H
4			✓		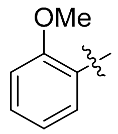				C	H
5			✓		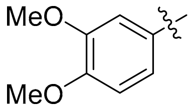				C	H
6			✓		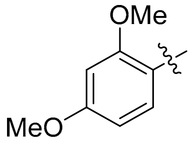				C	H
7			✓		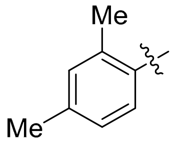				C	H
8			✓		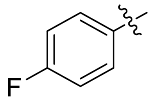				C	H

9		✓	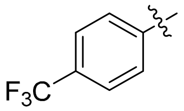			C	H
10		✓	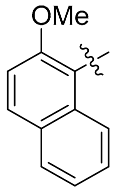			C	H
11		✓		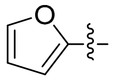		C	H
12		✓		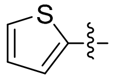		C	H
13	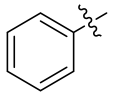				✓	C	H
14	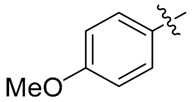				✓	C	H
15	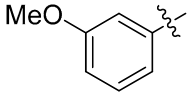				✓	C	H
16	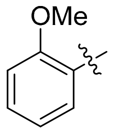				✓	C	H
17	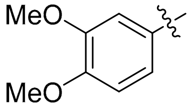				✓	C	H
18	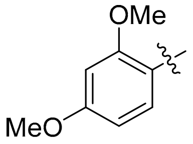				✓	C	H
19	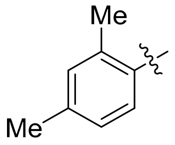				✓	C	H

20	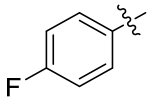			✓	C	H
21	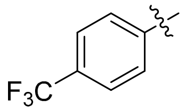			✓	C	H
22	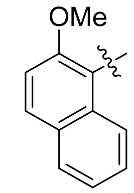			✓	C	H
23		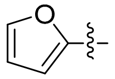		✓	C	H
24		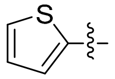		✓	C	H
25	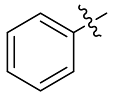		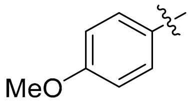		C	H
26	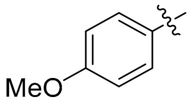		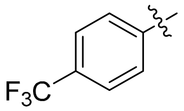		C	H
27	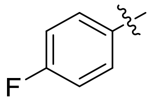		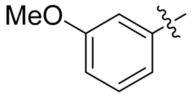		C	H
28	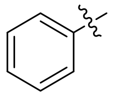		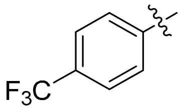		C	H
29	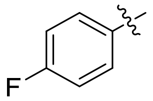		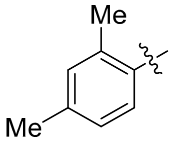		C	H
30	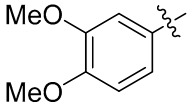		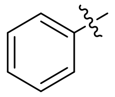		C	H

31	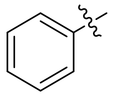			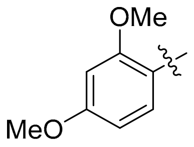			C	H
32	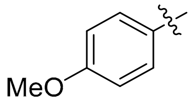			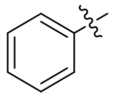			C	H
33	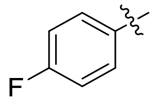				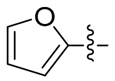		C	H
34		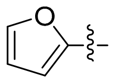		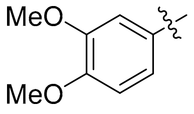			C	H
35	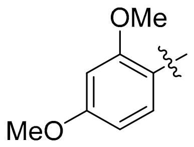				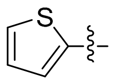		C	H
36		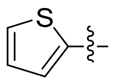		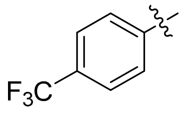			C	H
110			✓	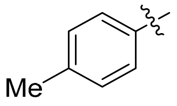			C	H
111			✓		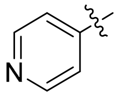		C	H
112			✓		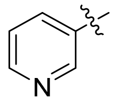		C	H
113	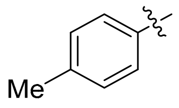					✓	C	H
114		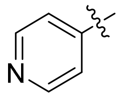				✓	C	H
115		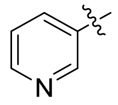				✓	C	H

116	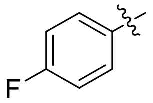			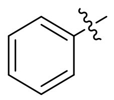		C	H
117	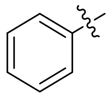				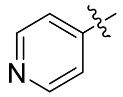	C	H
118	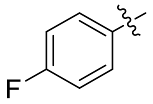				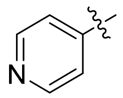	C	H
119		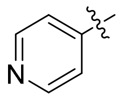		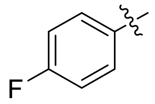		C	H
120		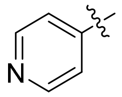			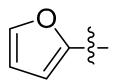	C	H
121		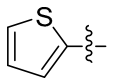			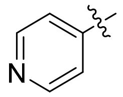	C	H
122			✓	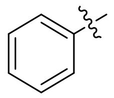		C	F
123			✓	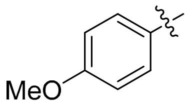		C	F
124			✓	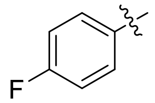		C	F
125			✓	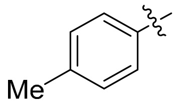		C	F
126			✓	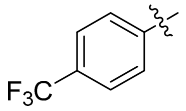		C	F
127			✓		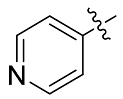	C	F

128			✓	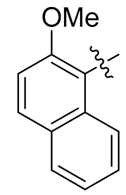		C	F
129			✓		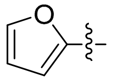	C	F
130			✓		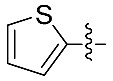	C	F
131	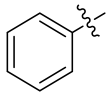			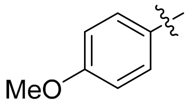		C	F
132	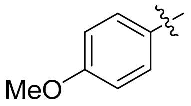			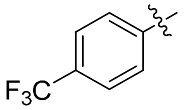		C	F
133	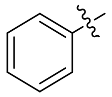			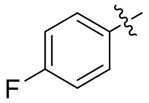		C	F
134	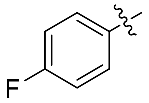				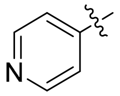	C	F
135	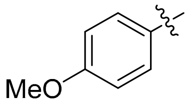			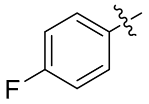		C	F
136	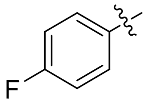				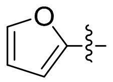	C	F
137		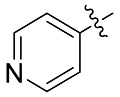		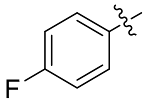		C	F
138		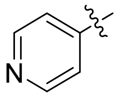			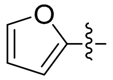	C	F
139		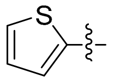			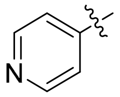	C	F

140		✓	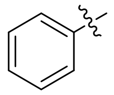		C	Cl
141		✓	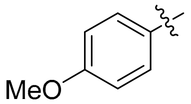		C	Cl
142		✓	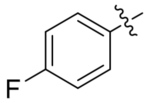		C	Cl
143		✓	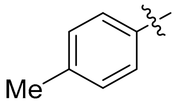		C	Cl
144		✓	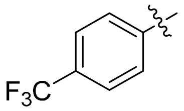		C	Cl
145		✓		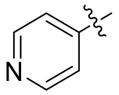	C	Cl
146		✓	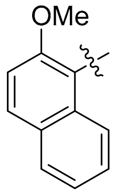		C	Cl
147		✓		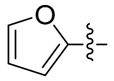	C	Cl
148		✓		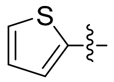	C	Cl
149	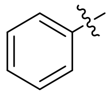		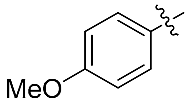		C	Cl
150	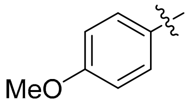		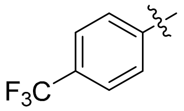		C	Cl
151	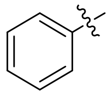		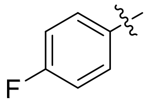		C	Cl

152	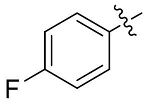				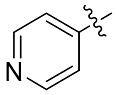	C	Cl
153	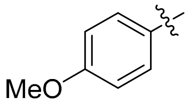			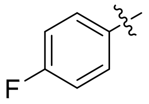		C	Cl
154	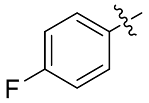				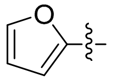	C	Cl
155		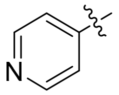		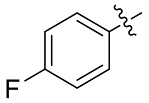		C	Cl
156		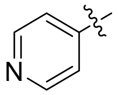			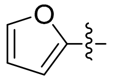	C	Cl
157		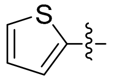			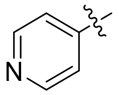	C	Cl
158			✓	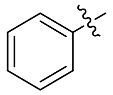		N	
159			✓	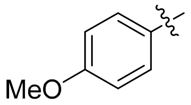		N	
160			✓	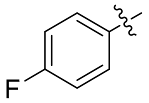		N	
161			✓	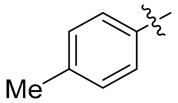		N	
162			✓	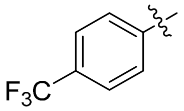		N	
163			✓		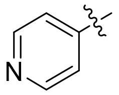	N	

164			✓	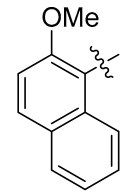		N
165			✓		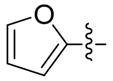	N
166			✓		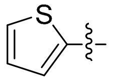	N
167	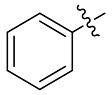			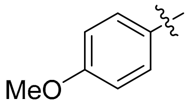		N
168	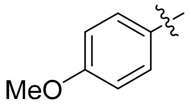			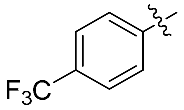		N
169	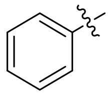			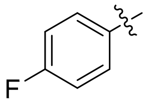		N
170	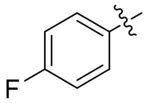				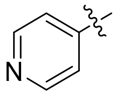	N
171	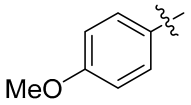			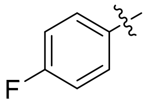		N
172	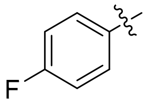				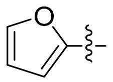	N
173		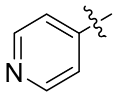		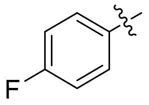		N
174		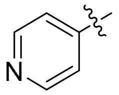			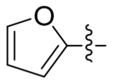	N
175		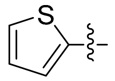			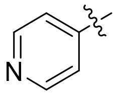	N

176		✓	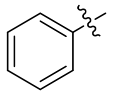	C	CF_3
177		✓	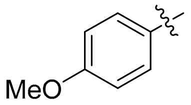	C	CF_3
178		✓	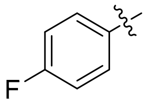	C	CF_3
179		✓	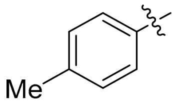	C	CF_3
180		✓	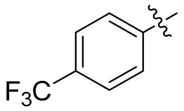	C	CF_3
181		✓	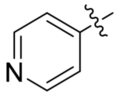	C	CF_3
182		✓	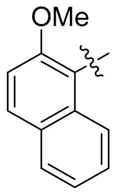	C	CF_3
183		✓	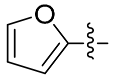	C	CF_3
184		✓	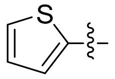	C	CF_3
185	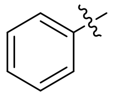		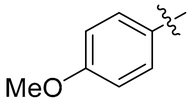	C	CF_3
186	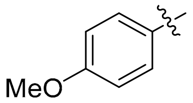		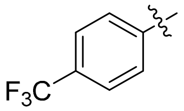	C	CF_3
187	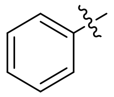		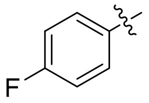	C	CF_3

188	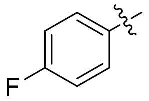				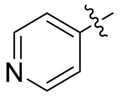	C	CF_3
189	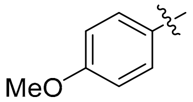			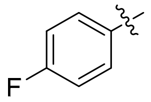		C	CF_3
190	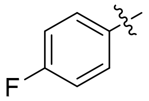				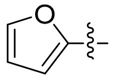	C	CF_3
191		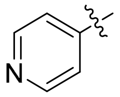		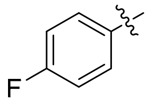		C	CF_3
192		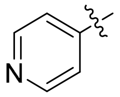			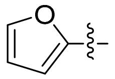	C	CF_3
193		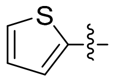			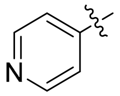	C	CF_3
194			✓	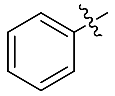		C	Morpholyl
195			✓	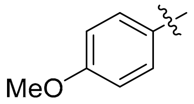		C	Morpholyl
196			✓	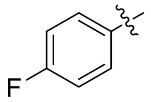		C	Morpholyl
197			✓	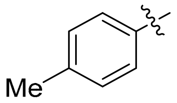		C	Morpholyl
198			✓	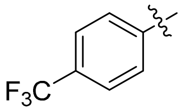		C	Morpholyl
199			✓		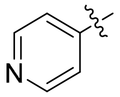	C	Morpholyl

200			✓	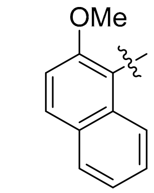		C	Morpholyl
201			✓		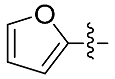	C	Morpholyl
202			✓		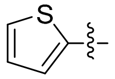	C	Morpholyl
203	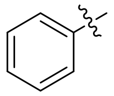			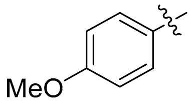		C	Morpholyl
204	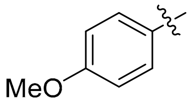			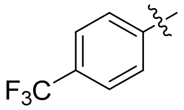		C	Morpholyl
205	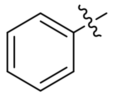			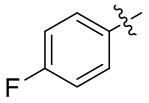		C	Morpholyl
206	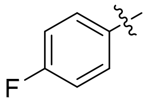				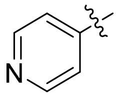	C	Morpholyl
207	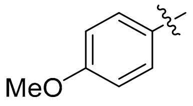			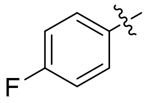		C	Morpholyl
208	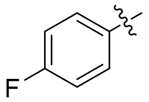				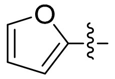	C	Morpholyl
209		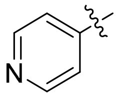		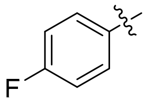		C	Morpholyl
210		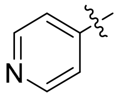			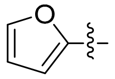	C	Morpholyl
211		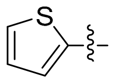			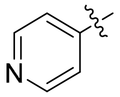	C	Morpholyl

212	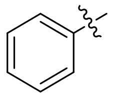			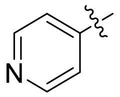	C	Morpholyl
213		✓	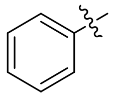		C	H
214		✓	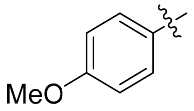		C	H
215		✓	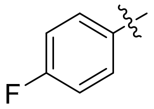		C	H
216		✓		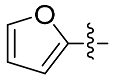	C	H
217		✓	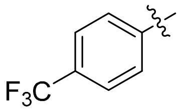		C	H
218		✓		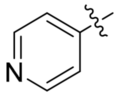	C	H
219		✓	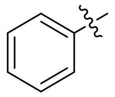		C	F
220		✓	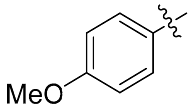		C	F
221		✓	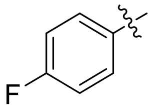		C	F
222		✓		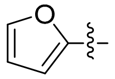	C	F
223		✓	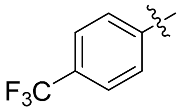		C	F
224		✓		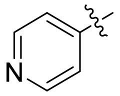	C	F

225	✓	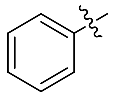		C	Cl
226	✓	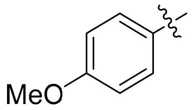		C	Cl
227	✓	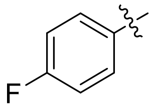		C	Cl
228	✓		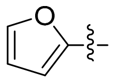	C	Cl
229	✓	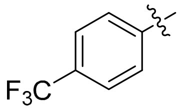		C	Cl
230	✓		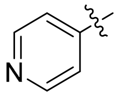	C	Cl
231	✓	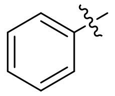		N	
232	✓	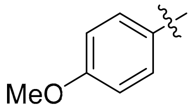		N	
233	✓	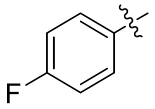		N	
234	✓		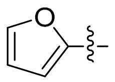	N	
235	✓	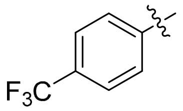		N	
236	✓		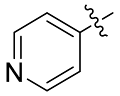	N	
237	✓	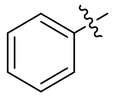		C	CF_3

238		✓	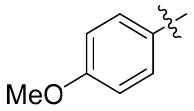	C	CF_3
239		✓	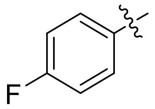	C	CF_3
240		✓	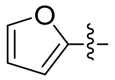	C	CF_3
241		✓	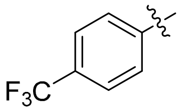	C	CF_3
242		✓	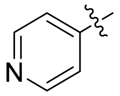	C	CF_3
243		✓	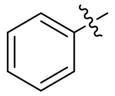	C	Morpholyl
244		✓	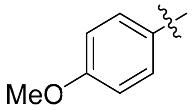	C	Morpholyl
245		✓	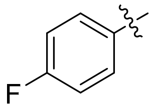	C	Morpholyl
246		✓	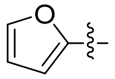	C	Morpholyl
247		✓	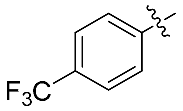	C	Morpholyl
248		✓	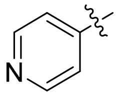	C	Morpholyl
249	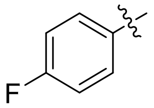		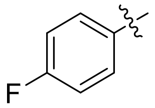	C	F

250	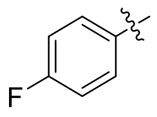	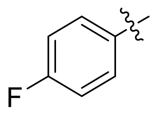	C	Phenyl

*The compounds that fall within the gap of this list belong to other chemical classes (flavanones and related flavones) and were not included in this study

Figure S2. Multiple sequence alignment of proteases. The sequences of the OPB protease (Lbrm_09.0850, A) and CPB protease (Lbrm_08.0830, B) were aligned with their respective templates, 2XE4 and 6PE4. The alignment showed 85.5% identity for OPB and 76.5% identity for CPB. The identity of residues is indicated by asterisks (*), while those with similar properties are marked by colons (:) or dots (.). The amino acid residues of the active site are indicated by red arrows (➔). The alignment was performed using Clustal Omega (https://www.ebi.ac.uk/jdispatcher/msa/clustalo).

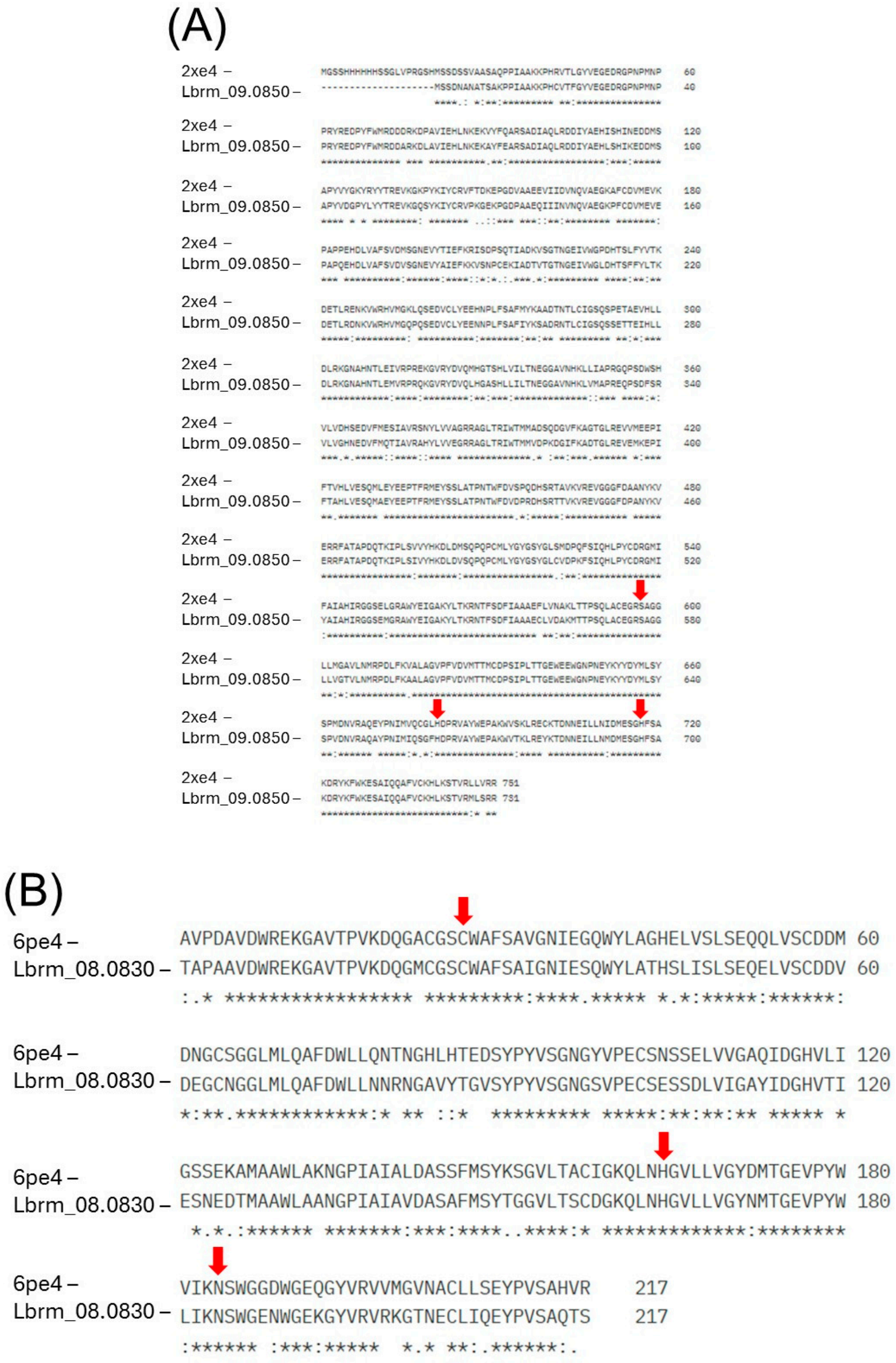

Figure S3. Ramachandran plot for the conformational analysis of OPB and CPB. In the diagram, the allowed regions for β-sheet and α-helix conformations are indicated. The ψ (psi) and φ (phi) torsion angles of the amino acid residues (blue) in OPB (A) and CPB (B) and are represented. Ramachandran plots to assess the stereochemical quality of the modeled 3D structure using the Swiss model validation server: OPB_Ramachandran Favoured 95.45% (OPB) and % 97.17(CPB); Ramachandran Outliers 0.28% (OPB) and 0.94% (CPB).

(A)

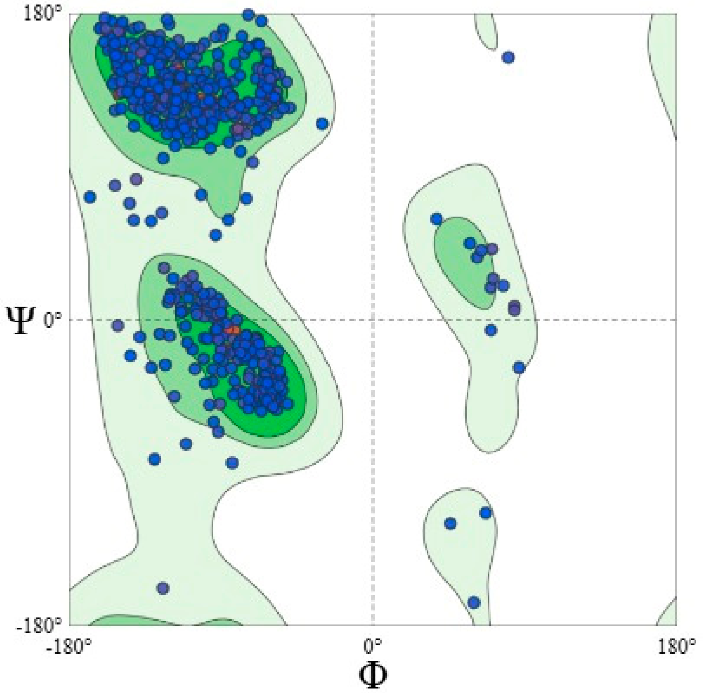

(B)

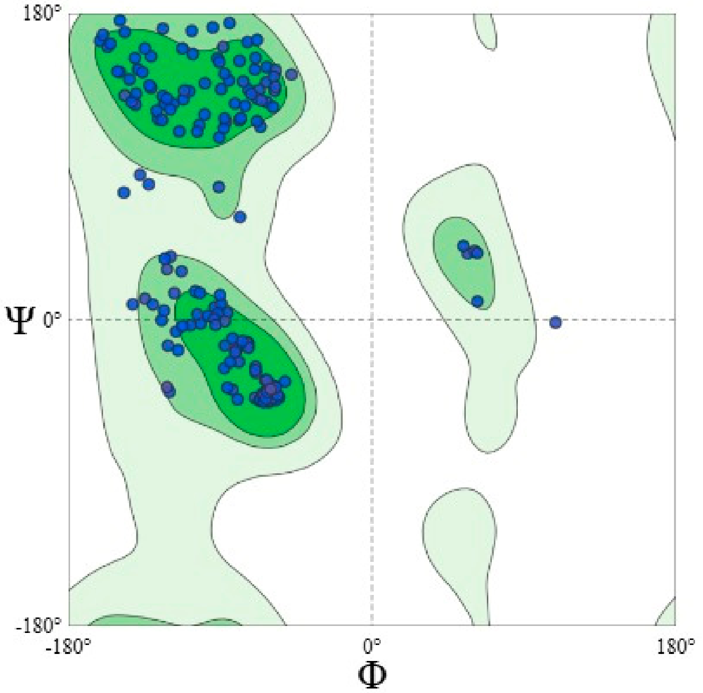

Figure S4. Molecular interaction of crystallographic references 2XE4 and 6P4E on *Leishmania (V.) braziliensis* proteinases. CPB (CYS25, HIS163 and ASN183) and OPB (SER568, ASP653, and HIS588) amino acids from catalytic site are indicated dotted circle. The representations of Antipain and GES interactions with proteinases are shown in 2D as a chemical structure

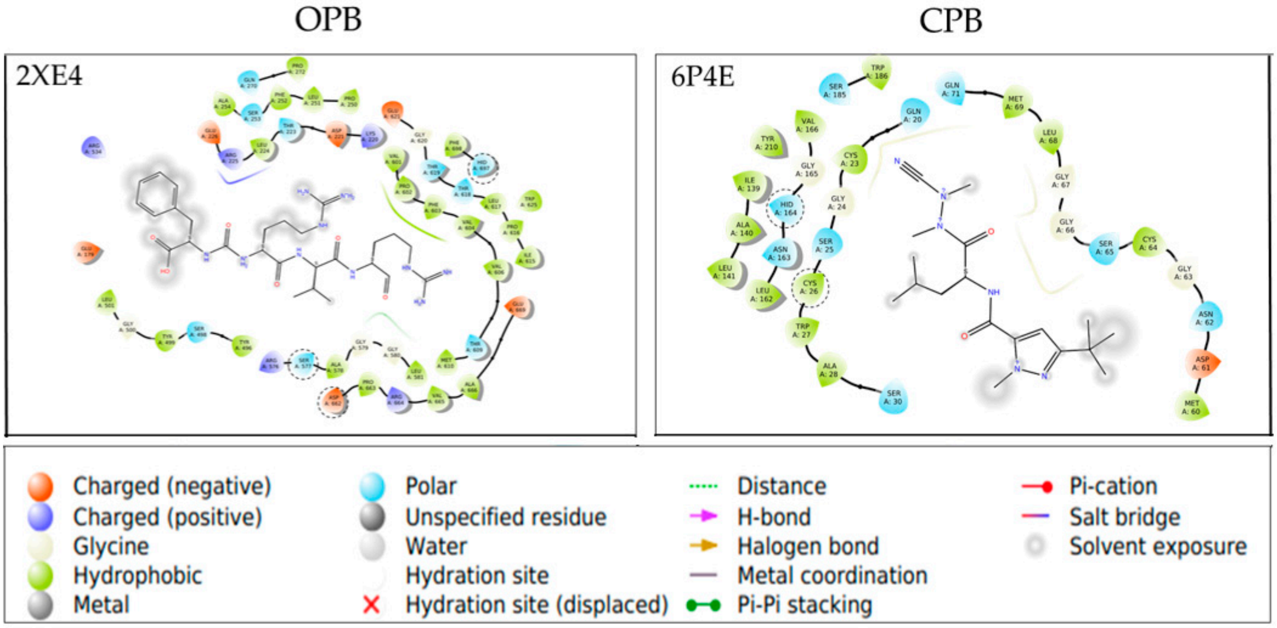

Table S2. Molecular screening of chalcone derivatives in the OPB and CPB of *L. (V.) braziliensis.* Chalcone derivatives were assessed for their binding potential to OPB and CPB proteinases. This approach was carried out using the Chalcone library (Table S1), and virtual screening experiments were performed using the Dockthor server. The results of the virtual screening for these compounds are presented in this table, organized by affinity scores.

Inhibitors	OPB		CPB	
	Compounds	Score (Kcal/mol)	Compounds	Score (Kcal/mol)
Crystallized	Antipain	-7.42	Antipain	-7.28
	GES	-7.53	GES	-8.10
Commercial	E-64	-6.66	E-64	-8.00
	PMFS	-6.59	PMFS	-7.13
Precursors	FKB	-7.87	FKB	-8.04
	C020	-7.99	C020	-6.66
Chalcone derivatives	C-132	-9.29	C-191	-9.09
	C-153	-9.22	C-128	-9.02
	C-26	-9.18	C-151	-8.97
	C-135	-9.09	C-223	-8.97
	C-250	-9.07	C-135	-8.97
	C-35	-9.04	C-185	-8.95
	C-249	-9.03	C-131	-8.94
	C-133	-9.03	C-168	-8.94
	C-25	-9.03	C-116	-8.87
	C-212	-8.97	C-155	-8.83
	C-191	-8.97	C-133	-8.83
	C-188	-8.96	C-28	-8.81
	C-131	-8.92	C-119	-8.81
	C-29	-8.90	C-229	-8.80
	C-31	-8.90	C-149	-8.80
	C-141	-8.89	C-180	-8.80
	C-186	-8.89	C-137	-8.78
	C-204	-8.89	C-189	-8.78
	C-27	-8.87	C-220	-8.76
	C-168	-8.86	C-173	-8.74
	C-209	-8.86	C-235	-8.74
	C-33	-8.85	C-215	-8.73
	C-32	-8.84	C-164	-8.72
	C-151	-8.83	C-30	-8.72
	C-229	-8.80	C-141	-8.71
	C-22	-8.79	C-167	-8.71
	C-190	-8.79	C-226	-8.71
	C-171	-8.78	C-182	-8.68
	C-244	-8.74	C-123	-8.68
	C-245	-8.74	C-120	-8.67
	C-28	-8.74	C-233	-8.67
	C-154	-8.72	C-187	-8.66
	C-243	-8.71	C-227	-8.64
	C-185	-8.71	C-214	-8.63
	C-116	-8.70	C-125	-8.62
	C-223	-8.69	C-169	-8.62
	C-149	-8.69	C-217	-8.61
	C-18	-8.69	C-27	-8.61
	C-150	-8.67	C-225	-8.60
	C-136	-8.63	C-219	-8.59
	C-19	-8.62	C-213	-8.58
	C-225	-8.61	C-231	-8.58
	C-248	-8.60	C-134	-8.57
	C-227	-8.59	C-121	-8.55
	C-206	-8.58	C-232	-8.55
	C-247	-8.58	C-250	-8.54
	C-205	-8.57	C-136	-8.54
	C-167	-8.57	C-156	-8.54

Chalcone derivatives	C-16	-8.55	C-249	-8.53
	C-239	-8.55	C-159	-8.53
	C-237	-8.55	C-192	-8.52
	C-134	-8.52	C-139	-8.52
	C-242	-8.52	C-247	-8.51
	C-192	-8.50	C-188	-8.50
	C-169	-8.50	C-239	-8.50
	C-14	-8.49	C-24	-8.50
	C-180	-8.49	C-146	-8.50
	C-226	-8.47	C-224	-8.50
	C-221	-8.47	C-20	-8.49
	C-152	-8.46	C-157	-8.47
	C-203	-8.45	C-161	-8.47
	C-144	-8.43	C-175	-8.47
	C-118	-8.43	C-16	-8.46
	C-156	-8.41	C-154	-8.46
	C-155	-8.41	C-118	-8.46
	C-219	-8.40	C-152	-8.45
	C-235	-8.40	C-138	-8.45
	C-208	-8.39	C-110	-8.44
	C-230	-8.39	C-230	-8.44
	C-189	-8.38	C-17	-8.44
	C-113	-8.38	C-14	-8.44
	C-137	-8.38	C-236	-8.44
	C-220	-8.36	C-221	-8.43
	C-215	-8.35	C-23	-8.43
	C-126	-8.35	C-10	-8.42
	C-30	-8.34	C-35	-8.41
	C-201	-8.34	C-143	-8.41
	C-238	-8.34	C-216	-8.40
	C-10	-8.33	C-153	-8.39
	C-213	-8.30	C-132	-8.39
	C-175	-8.30	C-158	-8.38
	C-138	-8.29	C-13	-8.38
	C-198	-8.27	C-04	-8.37
	C-139	-8.26	C-32	-8.37
	C-241	-8.26	C-234	-8.37
	C-157	-8.26	C-186	-8.37
	C-193	-8.26	C-36	-8.36
	C-146	-8.24	C-160	-8.36
	C-09	-8.24	C-241	-8.35
	C-172	-8.24	C-117	-8.34
	C-17	-8.23	C-122	-8.33
	C-207	-8.23	C-170	-8.33
	C-07	-8.23	C-237	-8.33
	C-173	-8.22	C-33	-8.33
	C-05	-8.22	C-142	-8.32
	C-222	-8.22	C-112	-8.30
	C-195	-8.22	C-177	-8.30
	C-162	-8.21	C-222	-8.30
	C-224	-8.20	C-238	-8.30
	C-231	-8.20	C-124	-8.29
	C-119	-8.19	C-172	-8.29
	C-216	-8.18	C-242	-8.27
	C-233	-8.18	C-178	-8.26
	C-228	-8.16	C-144	-8.25
	C-13	-8.14	C-228	-8.24
	C-218	-8.14	C-127	-8.23
	C-197	-8.12	C-145	-8.22
	C-121	-8.12	C-190	-8.22

Chalcone derivatives	C-214	-8.12	C-203	-8.21
	C-02	-8.11	C-218	-8.20
	C-21	-8.11	C-176	-8.20
	C-110	-8.10	C-111	-8.20
	C-166	-8.10	C-19	-8.19
	C-06	-8.07	C-179	-8.19
	C-200	-8.07	C-166	-8.18
	C-129	-8.06	C-21	-8.17
	C-24	-8.05	C-204	-8.15
	C-120	-8.05	C-05	-8.15
	C-236	-8.05	C-198	-8.13
	C-34	-8.04	C-150	-8.12
	C-183	-8.03	C-126	-8.11
	C-217	-8.03	C-163	-8.10
	C-234	-8.01	C-34	-8.10
	C-211	-8.00	C-22	-8.09
	C-20	-7.99	C-26	-8.08
	C-15	-7.99	C-15	-8.08
	C-182	-7.98	C-207	-8.07
	C-194	-7.96	C-183	-8.07
	C-174	-7.96	C-196	-8.06
	C-179	-7.95	C-171	-8.05
	C-246	-7.94	C-129	-8.05
	C-11	-7.93	C-174	-8.04
	C-36	-7.93	C-09	-8.04
	C-114	-7.91	C-195	-7.99
	C-115	-7.88	C-193	-7.98
	C-210	-7.87	C-165	-7.98
	C-240	-7.86	C-162	-7.96
	C-202	-7.86	C-240	-7.96
	C-128	-7.85	C-201	-7.95
	C-184	-7.84	C-130	-7.94
	C-125	-7.83	C-197	-7.94
	C-196	-7.83	C-31	-7.94
	C-164	-7.83	C-148	-7.94
	C-04	-7.83	C-194	-7.92
	C-170	-7.83	C-25	-7.92
	C-117	-7.82	C-114	-7.92
	C-181	-7.81	C-03	-7.92
	C-176	-7.79	C-212	-7.91
	C-178	-7.79	C-140	-7.91
	C-232	-7.79	C-29	-7.91
	C-158	-7.78	C-02	-7.88
	C-199	-7.78	C-06	-7.87
	C-143	-7.76	C-113	-7.87
	C-111	-7.76	C-18	-7.86
	C-163	-7.74	C-208	-7.85
	C-03	-7.71	C-184	-7.85
	C-160	-7.69	C-205	-7.82
	C-142	-7.69	C-211	-7.80
	C-159	-7.69	C-08	-7.79
	C-165	-7.64	C-244	-7.77
	C-130	-7.62	C-210	-7.74
	C-147	-7.62	C-115	-7.72
	C-140	-7.62	C-01	-7.67
	C-161	-7.62	C-12	-7.66
	C-145	-7.61	C-11	-7.65
	C-124	-7.60	C-07	-7.65
	C-148	-7.59	C-209	-7.64
	C-23	-7.59	C-200	-7.62
	C-123	-7.56	C-199	-7.62

C-122	-7.55	C-245	-7.58
C-127	-7.53	C-246	-7.54
C-12	-7.53	C-248	-7.53
C-187	-7.47	C-243	-7.49
C-08	-7.47	C-147	-7.43
C-01	-7.45	C-202	-7.42
C-177	-7.44	C-206	-7.39
C-112	-7.38	C-181	-7.13

Table S3. List of clusters and the respective components. Chalcone clustering components related to *L. (V.) braziliensis* proteinases were based on DataWarrior analysis. The OPB clusters (n = 7) are described as 'a' to 'g', and the CPB clusters (n = 9) as 'a' to 'i'.

OPB							
**Clusters**	**Cluster_a**	**Cluster_b**	**Cluster_c**	**Cluster_d**	**Cluster_e**	**Cluster_f**	**Cluster_g**
Centroids	C-191	C-250	C -153	C-31	C-212	C-132	C-35
Chalcones	C-188	C-29<br>C-151<br>C-249<br>C-133<br>C-116	C-149<br>C-32<br>C-25<br>C-135<br>C-131	C-10<br>C-04<br>C-06<br>C-128<br>C-05	C-203<br>C-207<br>C-205<br>C-206<br>C-209	C-26<br>C-185<br>C-189<br>C-150<br>C-186	C-130<br>C-148<br>C-12<br>C-166

CPB									
**Clusters**	**Cluster_a**	**Cluster_b**	**Cluster_c**	**Cluster_d**	**Cluster_e**	**Cluster_f**	**Cluster_g**	**Cluster_h**	**Cluster_i**
Centroids	C-185	C-223	C-155	C-191	C-28	C-151	C-180	C-119	C-135
Chalcones	C-189<br>C-132<br>C-186<br>C-150<br>C-25	C-198<br>C-204<br>C-217<br>C-229<br>C-235<br>C-237<br>C-238<br>C-239<br>C-241<br>C-242<br>C-247	C-152	C-04<br>C-05<br>C-06<br>C-10<br>C-31<br>C-128<br>C-188	C-187	C-12<br>C-29<br>C-35<br>C-116<br>C-130<br>C-133<br>C-148<br>C-166<br>C-167<br>C-171<br>C-249<br>C-250	C-09<br>C-126<br>C-144<br>C-176<br>C-177<br>C-178<br>C-179<br>C-182	C-117<br>C-118<br>C-134<br>C-137<br>C-169<br>C-170<br>C-173	C-25<br>C-32<br>C-131<br>C-149<br>C-153

Table S4. Physicochemical and ADMET properties of chalcones and inhibitors. Physicochemical parameters were assessed for 178 designed chalcones and the commercial inhibitors. The results are presented according to Lipinski's rules: no violations, one violation, two violations, and three violations. Data on molecular weight (MW), heavy atoms, aromatic heavy atoms, fraction of sp^3 carbon atoms (Fsp^3), rotatable bonds, hydrogen bond donors (HBD), hydrogen bond acceptors (HBA), and topological polar surface area (TPSA) are shown.

Lipinski Violations	Molecule	MW[a]	Heavy atoms	Aromatic heavy atoms	Fraction Csp3[b]	Rotatable bonds	HBAs[c]	HBDs[d]	TPSA[e]
	FKB	284.31	21	12	0.12	5	4	1	55.76
	C020	433.51	32	18	0.22	9	5	0	126.91
	C-223	471.44	34	18	0.19	6	7	1	49.77
	C-168	475.46	35	24	0.07	7	7	1	59.42
	C-119	395.43	30	24	0	5	4	1	50.19
	C-220	433.47	32	18	0.19	6	5	1	59
No violations	C-173	396.41	30	24	0	5	5	1	63.08
	C-235	454.44	33	18	0.2	6	7	1	62.66
	C-215	403.45	30	18	0.16	5	4	1	49.77
	C-164	411.45	31	22	0.08	6	5	1	68.65
	C-141	394.85	28	18	0.09	6	4	1	55.76
	C-167	407.46	31	24	0.04	6	4	1	59.42
	C-226	449.93	32	18	0.19	6	4	1	59

C-123	378.39	28	18	0.09	6	5	1	55.76
C-120	367.4	28	23	0	5	4	1	63.33
C-233	404.43	30	18	0.17	5	5	1	62.66
C-227	437.89	31	18	0.16	5	4	1	49.77
C-214	415.48	31	18	0.19	6	4	1	59
C-169	395.43	30	24	0	5	4	1	50.19
C-217	453.45	33	18	0.19	6	6	1	49.77
C-225	419.9	30	18	0.16	5	3	1	49.77
C-219	403.45	30	18	0.16	5	4	1	49.77
C-213	385.46	29	18	0.16	5	3	1	49.77
C-231	386.44	29	18	0.17	5	4	1	62.66
C-121	383.46	28	23	0	5	3	1	78.43
C-232	416.47	31	18	0.2	6	5	1	71.89
C-136	402.39	30	23	0	5	5	1	50.44
C-156	401.84	29	23	0	5	4	1	63.33
C-159	361.39	27	18	0.09	6	5	1	68.65
C-192	435.39	32	23	0.04	6	7	1	63.33

	C-139	401.45	29	23	0	5	4	1	78.43
	C-239	471.44	34	18	0.19	6	7	1	49.77
	C-24	336.4	24	17	0.05	5	3	1	74.77
	C-224	404.43	30	18	0.17	5	5	1	62.66
	C-20	348.37	26	18	0.05	5	4	1	46.53
	C-157	417.91	29	23	0	5	3	1	78.43
	C-161	345.39	26	18	0.09	5	4	1	59.42
	C-175	384.45	28	23	0	5	4	1	91.32
	C-16	360.4	27	18	0.09	6	4	1	55.76
	C-118	395.43	30	24	0	5	4	1	50.19
	C-138	385.39	29	23	0	5	5	1	63.33
	C-110	344.4	26	18	0.09	5	3	1	46.53
	C-230	420.89	30	18	0.17	5	4	1	62.66
	C-17	390.43	29	18	0.12	7	5	1	64.99
	C-14	360.4	27	18	0.09	6	4	1	55.76
	C-236	387.43	29	18	0.17	5	5	1	75.55
	C-221	421.44	31	18	0.16	5	5	1	49.77

C-23	320.34	24	17	0.05	5	4	1	59.67
C-10	410.46	31	22	0.07	6	4	1	55.76
C-35	442.53	32	23	0.07	7	4	1	84
C-216	375.42	28	17	0.17	5	4	1	62.91
C-158	331.36	25	18	0.05	5	4	1	59.42
C-13	330.38	25	18	0.05	5	3	1	46.53
C-04	360.4	27	18	0.09	6	4	1	55.76
C-234	376.41	28	17	0.18	5	5	1	75.8
C-160	349.36	26	18	0.05	5	5	1	59.42
C-117	377.43	29	24	0	5	3	1	50.19
C-122	348.37	26	18	0.05	5	4	1	46.53
C-170	396.41	30	24	0	5	5	1	63.08
C-237	453.45	33	18	0.19	6	6	1	49.77
C-33	384.4	29	23	0	5	4	1	50.44
C-112	331.36	25	18	0.05	5	4	1	59.42
C-177	428.4	31	18	0.12	7	7	1	55.76
C-222	393.41	29	17	0.17	5	5	1	62.91

C-238	483.48	35	18	0.22	7	7	1	59
C-172	385.39	29	23	0	5	5	1	63.33
C-242	454.44	33	18	0.2	6	7	1	62.66
C-228	409.86	29	17	0.17	5	4	1	62.91
C-127	349.36	26	18	0.05	5	5	1	59.42
C-145	365.81	26	18	0.05	5	4	1	59.42
C-203	491.58	37	24	0.16	7	4	1	59
C-218	386.44	29	18	0.17	5	4	1	62.66
C-111	331.36	25	18	0.05	5	4	1	59.42
C-19	358.43	27	18	0.12	5	3	1	46.53
C-166	337.39	24	17	0.05	5	4	1	87.66
C-05	390.43	29	18	0.12	7	5	1	64.99
C-198	483.48	35	18	0.22	7	7	1	59
C-163	332.35	25	18	0.05	5	5	1	72.31
C-34	426.46	32	23	0.07	7	5	1	68.9
C-22	410.46	31	22	0.07	6	4	1	55.76
C-15	360.4	27	18	0.09	6	4	1	55.76

C-183	388.34	28	17	0.1	6	7	1	59.67
C-196	433.47	32	18	0.19	6	5	1	59
C-171	425.45	32	24	0.04	6	5	1	59.42
C-129	338.33	25	17	0.05	5	5	1	59.67
C-174	368.38	28	23	0	5	5	1	76.22
C-195	445.51	33	18	0.22	7	5	1	68.23
C-193	451.46	32	23	0.04	6	6	1	78.43
C-165	321.33	24	17	0.05	5	5	1	72.56
C-162	399.36	29	18	0.09	6	7	1	59.42
C-240	443.42	32	17	0.21	6	7	1	62.91
C-201	405.44	30	17	0.21	6	5	1	72.14
C-130	354.39	25	17	0.05	5	4	1	74.77
C-197	429.51	32	18	0.22	6	4	1	59
C-148	370.85	25	17	0.05	5	3	1	74.77
C-194	415.48	31	18	0.19	6	4	1	59
C-114	331.36	25	18	0.05	5	4	1	59.42
C-03	360.4	27	18	0.09	6	4	1	55.76

	C-212	462.54	35	24	0.13	6	4	1	62.66
	C-140	364.82	26	18	0.05	5	3	1	46.53
	C-02	360.4	27	18	0.09	6	4	1	55.76
	C-06	390.43	29	18	0.12	7	5	1	64.99
	C-113	344.4	26	18	0.09	5	3	1	46.53
	C-18	390.43	29	18	0.12	7	5	1	64.99
	C-208	469.5	35	23	0.14	6	5	1	62.91
	C-184	404.4	28	17	0.1	6	6	1	74.77
	C-211	468.57	34	23	0.14	6	4	1	90.9
	C-08	348.37	26	18	0.05	5	4	1	46.53
	C-210	452.5	34	23	0.14	6	5	1	75.8
	C-115	331.36	25	18	0.05	5	4	1	59.42
	C-01	330.38	25	18	0.05	5	3	1	46.53
	C-12	336.4	24	17	0.05	5	3	1	74.77
	C-11	320.34	24	17	0.05	5	4	1	59.67
	C-07	358.43	27	18	0.12	5	3	1	46.53
	C-209	480.53	36	24	0.13	6	5	1	62.66

	C-200	495.57	37	22	0.19	7	5	1	68.23
	C-199	416.47	31	18	0.2	6	5	1	71.89
	C-245	488.55	36	18	0.28	6	5	1	62.24
	C-246	460.52	34	17	0.3	6	5	1	75.38
	C-248	471.55	35	18	0.29	6	5	1	75.13
	C-243	470.56	35	18	0.28	6	4	1	62.24
	C-147	354.78	25	17	0.05	5	4	1	59.67
	C-202	421.51	30	17	0.21	6	4	1	87.24
	C-206	480.53	36	24	0.13	6	5	1	62.66
	C-181	399.36	29	18	0.09	6	7	1	59.42
	C-191	463.42	34	24	0.04	6	7	1	50.19
	C-128	428.45	32	22	0.07	6	5	1	55.76
	C-151	428.88	31	24	0	5	3	1	37.3
One violation	C-135	442.45	33	24	0.04	6	5	1	46.53
	C-185	474.47	35	24	0.07	7	6	1	46.53
	C-131	424.46	32	24	0.04	6	4	1	46.53
	C-116	394.44	30	24	0	5	3	1	37.3

C-155	429.87	31	24	0	5	4	1	50.19
C-133	412.43	31	24	0	5	4	1	37.3
C-28	444.44	33	24	0.04	6	5	1	37.3
C-229	487.9	34	18	0.19	6	6	1	49.77
C-149	440.92	32	24	0.04	6	3	1	46.53
C-180	466.37	33	18	0.12	7	9	1	46.53
C-137	413.42	31	24	0	5	5	1	50.19
C-189	492.46	36	24	0.07	7	7	1	46.53
C-30	436.5	33	24	0.07	7	4	1	55.76
C-182	478.46	35	22	0.11	7	7	1	55.76
C-187	462.43	34	24	0.04	6	6	1	37.3
C-125	362.39	27	18	0.09	5	4	1	46.53
C-27	424.46	32	24	0.04	6	4	1	46.53
C-134	413.42	31	24	0	5	5	1	50.19
C-250	488.52	37	30	0.00	6	4	1	37.30
C-249	430.42	32	24	0.00	5	5	1	37.30
C-247	538.56	39	18	0.3	7	7	1	62.24

C-188	463.42	34	24	0.04	6	7	1	50.19
C-146	444.91	32	22	0.07	6	4	1	55.76
C-154	418.84	30	23	0	5	4	1	50.44
C-152	429.87	31	24	0	5	4	1	50.19
C-143	378.85	27	18	0.09	5	3	1	46.53
C-153	458.91	33	24	0.04	6	4	1	46.53
C-132	492.46	36	24	0.07	7	7	1	46.53
C-32	406.47	31	24	0.04	6	3	1	46.53
C-36	450.47	32	23	0.04	6	5	1	65.54
C-142	382.81	27	18	0.05	5	4	1	46.53
C-124	366.36	27	18	0.05	5	5	1	46.53
C-178	416.36	30	18	0.09	6	7	1	46.53
C-144	432.82	30	18	0.09	6	6	1	46.53
C-190	452.4	33	23	0.04	6	7	1	50.44
C-176	398.37	29	18	0.09	6	6	1	46.53
C-179	412.4	30	18	0.12	6	6	1	46.53
C-21	398.37	29	18	0.09	6	6	1	46.53

	C-126	416.36	30	18	0.09	6	7	1	46.53
	C-26	474.47	35	24	0.07	7	6	1	46.53
	C-207	509.57	38	24	0.16	7	5	1	59
	C-09	398.37	29	18	0.09	6	6	1	46.53
	C-31	436.5	33	24	0.07	7	4	1	55.76
	C-25	406.47	31	24	0.04	6	3	1	46.53
	C-29	422.49	32	24	0.07	5	3	1	37.3
	C-205	479.54	36	24	0.13	6	4	1	49.77
	C-244	500.59	37	18	0.3	7	5	1	71.47
Two violations	C-186	542.47	39	24	0.1	8	9	1	46.53
	C-241	521.45	37	18	0.22	7	9	1	49.77
	C-204	559.57	41	24	0.18	8	7	1	59
	C-150	508.92	36	24	0.07	7	6	1	46.53

[a]Molecular weight (MW); [b]Ratio of sp3 hybridized carbons over the total carbon count of the molecule (Fraction Csp3); [c]Hydrogen bond acceptors (HBAs); [d]H-bond donors (HBDs); [e]Topological polar surface area (TPSA)
